# Association Between Social Support and Job Satisfaction Among Mainland Chinese Ethnic Minority Kindergarten Teachers: The Mediation of Self-Efficacy and Work Engagement

**DOI:** 10.3389/fpsyg.2020.581397

**Published:** 2020-11-05

**Authors:** Shiyong Wu, Shuyi Zhou, Xiaoyan Yu, Wei Chen, Wen Zheng, Mingxi Huang, Hongbao Zhang, Xiujuan Li, Guangbao Fang, Xiaowei Zhao, Kai Zhang

**Affiliations:** ^1^South China Vocational Education Research Center, South China Normal University, Guangzhou, China; ^2^School of Education, South China Normal University, Guangzhou, China; ^3^School of Education, Huizhou University, Huizhou, China; ^4^Office of Humanities and Social Science, South China Normal University, Guangzhou, China; ^5^Faculty of Education, Monash University, Melbourne, VIC, Australia; ^6^Changan Center Kindergarten, Dongguan, China; ^7^College of Education, Huaibei Normal University, Huaibei, China

**Keywords:** social support, work engagement, self-efficacy, job satisfaction, ethnic minority, kindergarten teacher

## Abstract

Grounded on the social exchange theory (SET), social cognitive theory (SCT), and self-determination theory (SDT), this study aims to explore the mediating role of self-efficacy (SE) and work engagement (WE) on the effect of social support (SS) on job satisfaction (JS) among Chinese ethnic minority kindergarten teachers (KTs). The results show that: (1) SS has a directly significant effect on JS; (2) WE mediates the relationship between SS and JS; and (3) SE mediates the relationship between SS and WE. Although the mediation of SE on the relationship between SS and JS is not found, the posited multiple mediations of SE and WE on the relationship between SS and JS are totally accepted. The results reveal that SS mainland Chinese ethnic minority KTs received can not only have a direct effect on JS but also have an indirect impact through the one-path mediating role of WE and the chain mediating role of SE and WE. The results suggest that governments, supervisors, and sponsors should work together to provide ethnic minority KTs with more prioritized SS from both cultural psychology and financial material and opportunities for facilitating professional knowledge and skills in order to enhance their SE, inspire their WE, and eventually accumulate their JS.

## Introduction

Job satisfaction (JS) of kindergarten teachers (KTs) has attracted considerable attention from scholars, educators, and policymakers over the past decade (Moriarty et al., 2001; Klassen and Chiu, 2010; Ylitapio-Mäntylä et al., 2012; Gaias et al., 2018). The factors affecting KTs’ JS vary in context. For example, Bhamani (2012) debated that the factors determining JS of teachers teaching in early childhood schools in Pakistan are work-related social support (SS), including supervision, coworkers, promotion, rewards, nature of work, payment, benefits, communication, and operational conditions. Similarly, Abu Taleb (2013) concluded that factors affecting JS of KTs in Amman could be primarily categorized into two dimensions: work-related conditions, such as SS, work environment, and fringe benefit, and personal-related variables, such as belief [e.g., self-efficacy (SE)], attitude, and behavior (e.g., work engagement, WE). Furthermore, by conducting a meta-analysis from a holistic perspective, Massari (2015) summarized that the key factors influencing preschool teachers’ JS are structured as three levels: micro-level (e.g., teacher’ SE and WE); meso-level (e.g., SS from affiliation); and macro-level (e.g., educational systems and policies at regional, national, or international level).

In addition, a few studies have investigated the factors that predict the level of JS of Chinese KTs. For instance, Jiang et al. (2019) demonstrated that JS of mainland Chinese KTs could be predicted by SS from the organization, such as teacher empowerment and organizational climate, while Zhang and Wang (2018) reported that KTs with high SE in achieving ideal-self gain a sense of WE and JS. Moreover, Yang et al. (2018) confirmed that KTs in Taiwan with highly effective WE would get greater JS. Wong and Zhang (2014) illustrated that KTs in Hong Kong who perceived school SS in collegial support and professional promotion positively tend to show higher JS levels.

Contrary to the results of the abovementioned research, Wang Y. (2012) stated that the relationship between SS and JS is non-linear and in an inverse U shape, which means that when there are more SS for work, the JS will be improved; however, over a certain cut-off, the JS will decrease with the increase of the SS. Moreover, Duggleby et al. (2009), Kuru and Katsaras (2016), and Sulistyo and Suhartini (2019) reported that SE is excluded from the significant factors, including WE, SS, spiritual well-being, and others, that influence JS of KTs. In addition, Saragih and Margaretha (2013) revealed that employee’s perceived SS from an organization has an insignificant effect on WE that is found correlated with JS.

No doubt, these previous studies have greatly enriched our understanding of KTs’ JS and its antecedents observed. Teachers who are satisfied with their job are often provided with plenty of support from society, supervisors, fellows, and families and feel self-sufficient and engaged in involving work tasks. Effective teachers are essential for an accomplished educational system and a productive institute (Gkolia et al., 2014). However, it is uncertain, even contradictory in existing empirical studies mentioned above, and lacks holistic research of integrating the representative determinants affecting JS into a synthetic model. More importantly, despite many cross-national studies conducted, we know less about how JS and its determinants commonly identified in theoretical and empirical studies, such as SS, SE, and WE, interact within cross-ethnical groups. Specifically, we know less about what variables are the key determinants of JS, how they interact, and what the mechanisms are among KTs with ethnic minority identity.

To bridge this gap and better understand the interaction between SS, SE, WE, and JS, we sought to assess the roles of SE and WE as mediators in the association between SS and JS in a sample composed of mainland Chinese ethnic minority KTs, which provides a specific model of Chinese setting. Constructing the correlation between these four constructs from an ethnical perspective would provide a theoretical reference with a combined evaluation dimension and make a practical contribution to improving national prioritized ethnic policy, promoting educational development in ethnical areas, and facilitating work-related happiness and well-being of ethnic minority KTs, which has valuable implications for other multiple ethnic countries dealing with the same issues.

## Literature Review

### SS and JS

Social support is defined as “the perception that one is cared for, esteemed, and part of a mutually supportive social network” (Wills, 1991). SS may come from social and community ties (Taylor, 2011), superiors (Bruce and Blackburn, 1992; Vroom, 2008), peers (Chiaburu and Harrison, 2008), families, friends, and others (Sarason et al., 1990). SS has commonly been differentiated into “perceived SS” and “received SS” (Barrera, 1986; Vangelisti, 2009). Perceived SS (subjective support) refers to the perceived availability and adequacy of social connections, such as being valued, respected, and loved by others; received SS (objective support) focuses on the quantity and quality of the support given by society and organization, such as financial assistance and goods (Eagle et al., 2019).

The concept of JS is multidimensionally defined as what it means and how it impacts and enhances employees’ work performance (Dugguh and Dennis, 2014). Spector (1997) suggested that JS is simply the extent to which people like (satisfaction) or dislike (dissatisfaction) their jobs, involving whether or not the job physically and psychologically meets employees’ needs for the things provided by work, while Kalleberg (1977) argued that JS refers to an overall effective orientation on the part of individuals toward work roles that they are presently occupying. JS is a unitary concept, and individuals may be characterized by some sort of vaguely defined attitude toward their whole job situation.

According to social exchange theory (SET) (e.g., Gouldner, 1960; Blau, 1964), SS plays a pivotal role in predicting and facilitating employees’ JS (Ferguson et al., 2012; Zhang et al., 2015). When perceiving and receiving support from organizational superiors and peers, employees tend to reciprocate by liking their jobs and developing loyalty to their organization. By contrast, feeling non-supported probably leads employees to dissatisfaction with the job and reducing adherence to the organization (Lambert et al., 2016). Several empirical studies have shown that SS is positively related to JS among educators. McGinty et al. (2008) argued that the support that preschool teachers received from organizations and society has a positive association with job performance. Similarly, Bataineh (2009) concluded that family support is positively correlated with personal accomplishments among specific education teachers. Yuh and Choi (2017) reported that SS that childcare teachers received from managers and colleagues positively predicts their JS.

Based on previous studies, it can be predicted that SS ethnic minority KTs received should play a crucially decisive role in promoting their JS; hence, the following hypothesis was formulated:

Hypothesis 1: SS mainland Chinese ethnic minority KTs obtained has a significant and positive impact on their JS.

### SE as a Mediator

According to social cognitive theory (SCT), SE is initially defined as individuals’ beliefs about their ability to perform a specific behavior successfully (Bandura, 1977). SE has a substantial impact on an individual’s cognitive processes and performance, including decision-making, academic achievement, and behavioral motivation (Bandura, 1995; Cheung and Sun, 1999). Teacher SE refers to teachers’ perceptions of their capacities in managing classrooms, improving students’ learning (Short, 1992), and utilizing effective instructional strategies (Tschannen-Moran and Hoy, 2001). Previous studies have shown that teachers with low SE experience greater difficulties in teaching, higher levels of job-related stress (Betoret, 2006), and lower levels of JS (Klassen et al., 2009). In contrast, teachers with higher classroom management SE or higher instructional strategies SE have greater JS (Klassen and Chiu, 2010).

Social support is perceived as an essential aspect to enhance SE. Cross-sectional studies have supported that people’s SE has a significantly positive linkage with the SS they receive (Karademas, 2006; Wang et al., 2015; Kassis et al., 2019). For instance, Wang et al. (2015) examined the relationship between SS and SE among women psychiatrists. Their results revealed that participants with more SS had a higher level of SE than those with less SS. Evidence from teacher education research also discovered that pre-teachers’ perceived SE might be affected directly by received fellow students’ support, which is directly connected to higher levels of need satisfaction (Kassis et al., 2019).

In summary, the more SS KTs receive, the higher SE they recognize, and higher SE further relates to higher JS, suggesting that SE may serve as a mediator in the association between SS and JS. To investigate that prediction, we formulated the following hypothesis:

Hypothesis 2: SE plays a significant and positive mediating role in the relationship between SS and JS among mainland Chinese ethnic minority KTs.

### WE as a Mediator

Work engagement is defined as “a positive, fulfilling, work-related state of mind characterized by vigor, dedication, and absorption” (Schaufeli et al., 2002). Vigor refers to high levels of energy and mental resilience while working, the willingness to invest effort in one’s work, and persistence in the face of difficulties. Dedication refers to being highly involved in one’s work and experiencing a sense of significance, enthusiasm, inspiration, pride, and challenge. Absorption refers to fully concentrating and being happily engrossed in one’s work, such that time passes quickly, and one has difficulty detaching oneself from work. Teacher engagement has been conceptualized as a motivational construct comprising four dimensions (Klassen and Chiu, 2011): cognitive engagement, denoting vigor, persistence, and investment of attentional resources in teaching; emotional engagement, referring to teachers’ positive emotional responses to their work; and social engagement with students and colleagues, denoting teachers’ levels of care for, commitment to, and connection with students and colleagues.

Previous studies have shown that SS is significantly positively related to employee engagement (Wang and Eccles, 2012), leading to higher employee dedication and consequently accomplishment (Meijman and Mulder, 1998). For example, Moonen (2018) examined the relationship between SS and employee engagement among 234 employees in a high-tech company in the Netherlands, illustrating that SS is a positive antecedent of employee engagement and the relationship between SS and employee engagement is partially mediated by SE. Furthermore, Orgambidez-Ramos and de Almeida (2017) concluded that JS among Portuguese nursing staff is significantly predicted by WE and SS from both supervisors and fellows, implying that SS enhances the impacts of WE on their satisfaction. Similarly, Nasurdin et al. (2018) depicted that WE mediates the relationship between SS and JS among Malaysian nurses. Research on the consequences of teachers’ WE also disclosed that teacher’s efficacy and WE are positively related to JS (Høigaard et al., 2012).

Underpinning these works, we assume that the more SS KTs obtain, the more engaged they are, and resultantly, more engaged work leads to higher JS, indicating that WE may be seen as a mediator in the relationship between SS and JS; hence, the following hypothesis was tested:

Hypothesis 3: WE also plays a significant and positive mediating role in the relationship between SS and JS among mainland Chinese ethnic minority KTs.

### SE and WE

According to self-determination theory (SDT; Deci and Ryan, 1985), employee’s SE in beliefs about their work competence provides them with intrinsic motivation to enhance their WE, contributing to the promotion of JS (Carolyn and Paula, 2013). Empirical studies report that SE has a positive impact on WE (Salanova et al., 2010, 2011; Consiglio et al., 2016). SE is regarded not only as a driver fueling WE but also as an antecedent promoting WE (Schaufeli and Salanova, 2010). For instance, Xanthopoulou et al. (2007) examined the role of SE in predicting WE among employees of an electrical engineering and electronics company in the Netherlands. The results indicated that individuals with high levels of WE are highly self-efficacious. Similarly, Consiglio et al. (2016) pointed out that high-level SE leads employees to experience a fulfilling state of WE in which they are enthusiastic about their work and energic about their goals. Llorens et al. (2007) investigated the reciprocal relationship between SE and WE, stating that SE has a positive impact on the levels of engagement.

Following these studies, we posit that SE is directly related to WE, which subsequently improves job performance. Therefore, the following hypothesis was tested:

Hypothesis 4: SE predicts WE and further mediates the relationship between SS and JS among mainland Chinese ethnic minority KTs.

**FIGURE 1:**
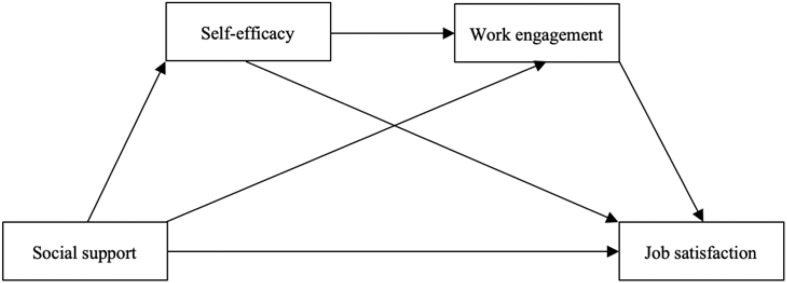
The posited model.

The complete hypothesized model is presented in Figure 1.

## Materials and Methods

### Participants

Participants comprised 207 teachers who work at kindergartens located in the underdeveloped ethnic areas of China, such as Xinjiang, Tibet, Inner Mongolia, Yunnan, Sichuan, Gansu, Guizhou, and Heilongjiang (Male = 22, Female = 185). All participants were recruited from a specific workshop sponsored by local governments and the state-owned enterprise of China Southern Air Holding Company, held by a top-tier teacher education university located in the developed coastal region of China, and taught by the research team consisting of three of the authors (SYW, XYY, and WC), with the obligations to provide poverty alleviation couplet-assistance aimed at promoting job skills and work achievement of ethnic minority KTs and eventually facilitating education equity regionally. Their ethnic identities included Mongol (94), Yi (21), Hui (18), Tibetan (15), Daur (14), Bai (12), Manchu (7), Miao (7), Uyghur (5), Tujia (4), Qiang (3), Zhuang (3), Buyi (1), Chinese Korean (1), Yao (1), and Russian (1). Almost 88.89% (*N* = 184) of participants come from state-run schools and the remainder (*N* = 23) work for private-run schools. Ages of participants ranged from 20 to 50 years (*M* = 26.47, *SD* = 6.85). More detailed demographical information about their degrees, experiences, and contracts can be found in Table 1.

**TABLE 1:** Demographical information of participants.

**Characteristics**	**Number**	**Percentage**
Gender		
Male	22	10.63%
Female	185	89.37%
Age		
20–30	107	51.69%
31–40	75	36.23%
41–50	19	9.18%
Over 50	6	2.9%
Affiliation		
Public	184	88.89%
Private	23	11.11%
Degree		
M.A.	10	4.83%
B.A.	153	73.91%
College	41	19.81%
Associate	3	1.45%
Experience		
1–5	104	50.24%
6–10	47	22.71%
11–20	32	15.46%
Over 20	24	11.59%
Title		
Associate professor	1	0.48%
Senior	24	11.59%
Third-grade teacher	9	4.35%
Second-grade teacher	62	29.95%
First-grade teacher	40	19.33%
Non-title	71	34.30%
Contract		
5 years	78	37.68%
Less than 5 years	129	62.32%

### Instruments

Social support was measured with ten items from the Chinese version of the Social Support Rating Scale (SSRS). The SSRS was originally developed by Cauce et al. (1982) with the intention to examine individual support received in society across three subscales: family support, formal support, and informal support (Stromborg and OIsen, 2004). The Chinese version of SSRS was revised by Xiao (1994, 1999) with the similarity of three dimensions, subjective support, objective support, and support utilization, and has been commonly used as a measure assessing SS in China (Xiao et al., 2017). The total support score is the sum from the three subscales. A higher score represents participants’ higher levels of SS. The test–retest reliability of the Chinese version of SSRS has exceeded 0.92, and the Cronbach’s alpha coefficient can be as high as 0.91 (Xiao, 1994, 1999). In this study, the Cronbach’s alpha coefficient is 0.67.

Job satisfaction was measured with the Chinese version of Spector’s (1985) Job Satisfaction Survey (JSS), a 36-item measure that examines how people feel about their job in a combination of psychological, physiological, and environmental circumstances (Hoppock, 1935; Spector, 1997). The Chinese version of the JSS was revised by Yang et al. (2010) and involves nine subscales: payment, promotion, supervision, fringe benefits, contingent rewards, operating conditions, coworkers, nature of work, and communication. Responses were solicited with a six-point Likert-type scale (1 = very much disagree, 6 = very much agree). The Cronbach’s alpha reliability coefficients of the original version range from 0.60 for the coworker’s subscale to 0.91 for the total scale; the 18-month test–retest reliability coefficients range from 0.37 for benefits to 0.74 for the total (Spector, 1997). The Cronbach’s alpha reliability coefficients of the Chinese version of the JSS range from 0.41 for the operating condition subscale to 0.91 for the total scale. In the present study, the Cronbach’s alpha reliability coefficients range from 0.55 for the promotion subscale to 0.93 for the total scale.

Self-efficacy is measured with the Chinese version of the General Self-efficacy Scale (GSES), a 10-item measure that examines an individual’s self-belief in difficulty or dilemma (Jerusalem and Schwarzer, 1992; Zhang and Schwarzer, 1995; Schwarzer et al., 1997a). Responses were solicited with a four-point Likert-type scale (1 = absolutely incorrect, 4 = absolutely correct). The Cronbach’s alpha reliability coefficients of the original version range from 0.75 to 0.91 (Schwarzer et al., 1997b, 1999), and that of the Chinese version can be as high as 0.92 (Zhang and Schwarzer, 1995; Cheung and Sun, 1999). In the present study, the Cronbach’s alpha reliability coefficient is 0.94.

Work engagement is measured with the Chinese version of the Utrecht Work Engagement Scale (UWES) (Schaufeli et al., 2002), a 17-item measure that assesses an individual’s positive, fulfilling, work-related state of mind (Schaufeli et al., 2001). It similarly consists of three dimensions: vigor, dedication, and absorption. Responses were collected with a seven-point Likert-type scale (0 = never, 7 = always). The Cronbach’s alpha reliability coefficients of the original version range from 0.80 to 0.90 (Schaufeli and Bakker, 2004a). The Chinese version of the UWES also has good reliability coefficients from 0.90 to 0.94 (Zhang and Gan, 2005; Gan et al., 2011). In this study, the Cronbach’s alpha reliability coefficient is 0.95.

### Procedure

Before conducting this research, we developed a course on educational research methods to impart the participants the knowledge and skills about how to design and respond a survey in order to obtain high-quality data because of the particularity and rarity of the samples. For the convenience of investigation, we merged the items of four measures into a single sheet, which was inputted into the online survey software Wenjuanxing, known as Chinese Qualtrics, so that participants could respond to them anywhere. Before distributing the survey form, participants were initially provided critical ethics approval and an explanatory statement informing them that they could freely decide whether or not to participate in this research. During the study from 3 August 2019 to 3 September 2019, we delivered the questionnaire link to the participants so that they could fill in online. After participants completed the survey, the data were collected and checked whether there were random or incongruent responses. Benefit from the well-prepared work, we obtained highly valid data from all the participants who enrolled in this workshop (*N* = 207).

### Data Analysis

We deployed SmartPLS version 3.3.2, the most widely used partial least square (PLS) statistical analysis software, to analyze the data. First, we evaluated the psychometric properties of the scales described in Section “SE as a Mediator” by testing reliability, convergent validity, and discriminant validity of the measurement model using confirmatory factor analysis. To improve the model’s satisfactory level, we retained the items with factor loading up to 0.7 for further analysis (Hair et al., 2013). Consequently, we ran the PLS Algorithm and the bootstrapping procedure with 5000 subsamples to compute the structural model and test the hypotheses. The mediation roles were estimated using the path coefficients and the level of significance (*t*-values) (Hair et al., 2012).

## Results

### Descriptive Statistics

Table 2 shows the means, standard deviations, and significant differences of the partly selected variables, affiliations, and ages, that are regarded as the essential indicators, from the organizational level and individual level, influencing JS (Abu Taleb, 2013). Regarding affiliation differences, except for SS, participants working at the public schools (vs. private schools) scored higher for SE, WE, and JS, but there are no significant differences among observed variables by schools. In terms of age, participants over 50 years old (vs. younger participants) had the highest scores for each variable, while the youngest participants between 20 and 30 years old scored lowest for SS, SE, and WE, but the difference was significant only for SE (*p* = 0.03).

**TABLE 2:** Means, standard deviation, and significant differences of variables of affiliation and experience.

**Variables**	***M***	***SD***	**Public school (*n* = 184)**		**Private school (*n* = 23)**		***t***	***Sig.***	**20–30 (*n* = 107)**		**31–40 (*n* = 75)**		**41–50 (*n* = 19)**		**Over 50 (*n* = 6)**		***F***	***Sig.***
			***M***	***SD***	***M***	***SD***			***M***	***SD***	***M***	***SD***	***M***	***SD***	***M***	***SD***		
SS	2.78	0.48	2.78	0.48	2.79	0.47	0.11	0.91	2.69	0.48	2.86	0.48	2.91	0.42	2.97	0.42	2.59	0.06
SE	2.96	0.67	2.98	0.69	2.82	0.56	1.05	0.30	2.88	0.65	3.00	0.65	3.12	0.79	3.58	0.58	2.93	0.03*
WE	5.30	1.00	5.31	1.01	5.22	0.93	0.36	0.72	5.23	1.04	5.24	0.96	5.49	0.99	5.59	0.94	0.49	0.69
JS	3.89	0.84	3.89	0.87	3.87	0.67	0.10	0.92	3.92	0.85	3.83	0.82	3.79	0.95	4.02	0.88	1.24	0.30

*N* = 207. SS, social support; SE, self-efficacy; WE, work engagement; JS, job satisfaction. *p < 0.05

Table 3 displays the correlations of the variables investigated and the Cronbach’s alpha reliability coefficients of the scales. The correlation is positively significant among SS, SE (*r* = 0.20; *p* < 0.01), WE (*r* = 0.23; *p* < 0.01), and JS (*r* = 0.26; *p* < 0.01). Teachers with a high level of SE also obtained high scores for WE (*r* = 0.38; *p* < 0.01). However, the association between SE and JS was found to be non-significant (*r* = 0.09; *p* > 0.05). Moreover, although the Cronbach’s alpha coefficient of SS is 0.67, it can be acceptable (Taber, 2018).

**TABLE 3:** Correlations of variables.

**Variables**	**1**	**2**	**3**	**4**	***M***	***SD***	***Skewness***	***Kurtosis***
SS	(0.67)				2.78	0.48	–0.25	–0.43
SE	0.20**	(0.94)			2.96	0.67	0.02	–0.87
WE	0.23**	0.38**	(0.95)		5.30	1.00	0.09	–0.73
JS	0.26**	0.09	0.30**	(0.93)	3.89	0.84	0.18	–0.32

*N* = 207, Cronbach’s alpha coefficients are presented in parentheses diagonally. SS, social support; SE, self-efficacy; WE, work engagement; JS, job satisfaction. **p < 0.01.

### Measurement Model Test

According to Roldán and Sánchez-Franco (2012), the measurement of model evaluation requires four aspects to be analyzed: factor loadings, composite reliability, Cronbach’s alpha, and average variance extracted (AVE). The threshold values are recommended to be greater than 0.7, 0.7, 0.7, and 0.5 for factor loadings (Carmines and Zeller, 1979), composite reliability, Cronbach’s alpha, and AVE, respectively (Fornell and Larcker, 1981). Table 4 presents the threshold values for each indicator. Factors with values over 0.7 were extracted as observed variables. The internal consistency reliabilities of latent variables range from 0.87 to 0.94 for Cronbach’s alpha and from 0.91 to 0.95 for composite reliabilities. The values of AVE also exceed the 0.5 cut-off threshold. Therefore, the modified measurement model shows adequate convergent validity.

**TABLE 4:** Factor loadings, Cronbach’s alpha, composite reliability, and average variance extracted.

**Construct**	**Items**	**Factor loading**	**Cronbach’s alpha**	**Composite reliability**	**Average variance extracted**
SS	Objective support 2	0.93	0.87	0.94	0.88
	Objective support 3	0.95			

SE	Self-efficacy 2	0.72	0.94	0.95	0.68
	Self-efficacy 3	0.75			
	Self-efficacy 4	0.84			
	Self-efficacy 5	0.86			
	Self-efficacy 6	0.79			
	Self-efficacy 7	0.86			
	Self-efficacy 8	0.85			
	Self-efficacy 9	0.88			
	Self-efficacy 10	0.88			

WE	Vigor 2	0.73	0.94	0.95	0.67
	Vigor 3	0.81			
	Absorption 3	0.81			
	Absorption 4	0.86			
	Absorption 5	0.78			
	Dedication 1	0.84			
	Dedication 2	0.85			
	Dedication 3	0.86			
	Dedication 4	0.81			

JS	Benefit 3	0.80	0.88	0.91	0.58
	Payment 1	0.75			
	Promotion 2	0.76			
	Rewards 1	0.76			
	Nature of work 2	0.74			
	Nature of work 3	0.75			
	Nature of work 4	0.78			

**N* = 207. SS, social support; SE, self-efficacy; WE, work engagement; JS, job satisfaction.*

Discriminant validity is also an important indicator for testing the adequacy of the measurement model. The SmartPLS 3 software provides the heterotrait–monotrait (HTMT) ratio to establish the discriminant validity. The values of the HTMT ratio should be less than 0.85 (Henseler et al., 2015). Table 5 reports the HTMT ratios for each pair construct with less than the recommended value. Hence, each latent variable is valid and displays high distinction from the other latent variables.

**TABLE 5:** Heterotrait–monotrait (HTMT) discrimination validity.

**Variables**	**1**	**2**	**3**	**4**
SS	–			
SE	0.14	–		
WE	0.08	0.36	–	
JS	0.09	0.37	0.59	–

**N* = 207. SS, social support; SE, self-efficacy; WE, work engagement; JS, job satisfaction. All the values are less than 0.85.*

### Structural Model Test

The structural model’s assessment involves the goodness of fit (GoF) indices, path coefficient, and *t*-values. The SmartPLS 3 provides a current statistical criterion to test the GoF, including standardized root-mean-square residual [SRMR, <0.1 suggested by Kline (2015)], d_ULS and d_G [>0.05 recommended by Dijkstra and Henseler (2015)], normed fit index [NFI, >0.8 recommended by Hooper et al. (2008)], and RMS_Theta [<0.12 recommended by Henseler et al. (2014)]. The results in Table 6 show that the model has an appropriate model fit index.

**TABLE 6:** Model fit index.

**Fit index**	**SRMR**	**d_ULS**	**d_G**	**NFI**	**RMS_Theta**
Proposed value	<0.10	>0.05	>0.05	>0.80	<0.12
Estimated value	0.07	1.76	0.88	0.87	0.10

**N* = 207. SRMR, standardized root mean-square residual; NFI, normed fit index.*

**FIGURE 2:**
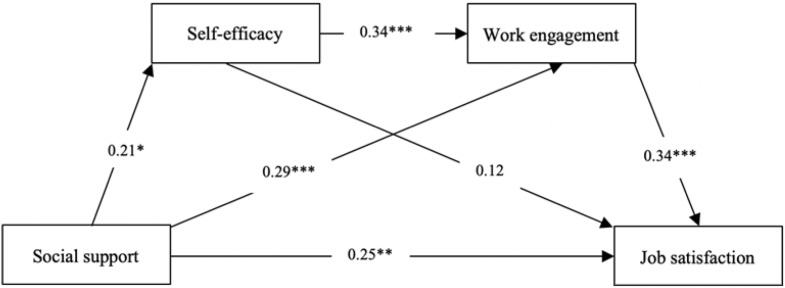
Model result.

The path coefficient beta values [greater than the threshold of 0.2 proposed by Chin (1998)] indicate the direct influence of the predictor upon the predicted latent constructs; the value of the t-statistics between each pair construct should be greater than 1.96. Figure 2 and Table 7 display the results of the model fit and hypothesis test.

**TABLE 7:** Result of the hypothesis test.

**Path**	**Coefficient**	***t***	***Hypothesis***
SS –>SE	0.21	2.43*	Supports
SS –> WE	0.29	3.84***	Supports
SS –> JS	0.25	3.17**	Supports
SE –> WE	0.34	5.01***	Supports
SE –> JS	0.12	1.43	Does not support
WE –> JS	0.34	4.90***	Supports

*N* = 207. SS, social support; SE, self-efficacy; WE, work engagement; JS, job satisfaction. *p < 0.05, **p < 0.01, ***p < 0.001.

Regarding H1, we posited that SS has a significant and positive impact on JS. The result indicates that SS has a significant direct predictive power for JS (β = 0.25, *p* < 0.01). Thus, H1 is fully supported.

In H2, we proposed that SE significantly mediates the association between SS and JS. The results indicate that there is a relatively significant effect of SS on SE (β = 0.21, *p* < 0.05), but the significant relationship between SE and JS is not supported as the path coefficient value is found to be β = 0.12 (*p* > 0.1). The overall indirect effect of SS → SE → JS is not significant [β = 0.03, *SE* = 0.01, 95% CI (−0.02, 0.08)]. Therefore, H2 is not supported.

Regarding H3, we proposed that WE plays a significant role in mediating the relationship between SS and JS. The results indicate that the path coefficients are significant both for SS to WE (β = 0.29, *p* < 0.001) and for WE to JS (β = 0.34, *p* < 0.001). The overall indirect effect of path SS → WE → JS is positively significant [β = 0.10, *SE* = 0.03, 95% CI (0.04, 0.18)]. Thus, H3 is fully supported.

In H4, we posited that SE predicts WE and further mediates the relationship between SS and JS. The results indicate that SE has a significant impact on WE (β = 0.34, *p* < 0.001), and the indirect effect of SS → SE → WE → JS is significant [β = 0.02, *SE* = 0.01, 95% CI (0.01, 0.05)]. The total indirect effect of SS on JS is also significant [β = 0.15, *SE* = 0.04, 95% CI (0.07, 0.25)]. Therefore, H4 is totally supported.

## Discussion

The present study aimed to investigate the mediating role of SE and WE on the effect of SS on JS among mainland Chinese ethnic minority KTs. In general, participants have upper-middle levels in SE, WE, and JS, but they obtain relatively low scores in SS, which supports the result of Wang L. (2012) who argued that ethnic monitory teachers in primary and secondary schools in western and northern areas are dissatisfied with SS available, payment, and workload. It is also congruent with the finding of Li and Li (2020) who reported that Chinese KTs perceived less organizational support. In fact, China’s government has implemented a series of prioritized policies to support ethnic monitory KTs’ professional promotion, career development, and life satisfaction since 1979. At the state level, some initiatives stipulate that ethnic minority teachers should be paid higher salary than local civil servants and teachers with Han identity, allocated additional subsidies for work and transportation, and involved in free skill training plan. More attractively, when ethnic monitory KTs’ children apply for high school and university, they can obtain entrance admission with scores far lower than those of Han candidates (Wei, 2009). At the local level, some ethnic monitory provinces have put a wide of more specifically preferential policies into force. For example, in Inner Mongolia, graduates with ethnic identities of Daur, Ewenki, Oroqen, and Mongol are given the priority to be recruited for KTs (He, 2018), while in Yunnan, at least one of ethnic monitory KTs should be appointed to school leadership and the ratio of senior and associate professor should be distributed to be 10% higher than that of other schools (Yunnan, 2013). However, ethnic minority KTs are not sensitive to these prioritized treatments and do not perceive these SS as a dominant stimulus for fulfilling their work task. This opposite result may be attributed to the big differences in value of SS between Han and other ethnic minorities. For example, Bai tribe values religion as the core of culture and criterion for the life, work, and communication (Yu, 2018). Mongol tribe with the mainstream of grassland culture respects clothing, games, ecological environment, and other ethnic events (Pu, 2018). Hui tribe integrating Islam and Han is characterized by highly appreciating healthy natural and social environment (Li, 2007). This preference of value that cherishes inherent well-being in psychology and spirituality rather than external support in material (Ding, 2012) can lead to feel a little unsupported.

Despite low level of SS, the empirical result shows that SS has a significant positive effect on JS, fully supporting the first hypothesis (H1). This result is consistent with the theoretical arguments in the literature (Ferguson et al., 2012; Zhang et al., 2015; Lambert et al., 2016; Almeida et al., 2019) and previous empirical findings (McGinty et al., 2008; Bataineh, 2009; Yuh and Choi, 2017). According to the SET, when receiving SS from organizations, employees may feel indebted and act reciprocatively engaged, contributing to high level of JS (Colquitt et al., 2013). Evidence was also found in this study that mainland Chinese ethnic minority KTs from both public and private schools who receive significant SS tend to have a high level of JS. Based on these findings, it can be inferred that SS is a vital construct for JS that should be considered when aspiring to improve levels of JS. Thus, central and local governments, educational administration departments, social sponsors, and kindergarten institutes need to take into account the importance of SS from cultural psychology because JS of ethnic minority KTs could be only achieved well when their internal target was supported well. They also need to consider the dynamics and diversities of SS, such as by increasing the additional work allowance in remote ethnic areas, raising wages, expanding rapid professional promotion avenues, improving the work environment, and ameliorating the barriers that would be detrimental to the promotion of JS regardless of the feature of their affiliations.

Another result reveals that despite the significant effect of SS on SE, the mediation role of SE on the relationship between SS and JS is not found, partly rejecting the second hypothesis (H2). Unlike the previous findings reporting that SE has a significant effect on JS (Lai and Chen, 2012; Borgogni et al., 2013), this result from the current research provides an opposite argument that the mainland Chinese ethnic minority KTs’ belief in SE has no effect on their JS. However, this result supports the findings concluded by Sulistyo and Suhartini (2019), who reported that there is no significant positive impact of SE toward JS among educators in Indonesia. This result is also in line with the research of Duggleby et al. (2009) and Kuru and Katsaras (2016), stating that SE does not have a significant effect on JS among Canadian and Greek nurses, respectively. It can also be evidenced that participants gain low scores in SS and there is a significant difference occurred in SE for the age variable. This result may be explained by the SCT that assumes that when employees lack confidence in their ability to fulfill work inspiration or lack opportunity and support to make knowledge contribution they will feel unaccomplished and dissatisfied with jobs. In order to reveal the insignificant result of SE toward JS of the participants, we supplemented the qualitative data obtained through randomly interviewing some of respondents. From the interview result, the interviewees complained that although being granted to teach in their ethnic language in classroom, they have no authorization to freely develop textbook and design curriculum completely in line with their traditional culture, which restricts their advantages in teaching ethnic-related contents and makes them depressed and helpless. Besides, they also felt unconfident in degree and skill. Compared to their Han tribe colleagues, most of them obtained their degrees through distance education program in part time and in lack of systematically professional training, which make them feel incompetent to the high requirement on creative and innovative education. Therefore, despite a gap between the assumed path and the empirical model, the providers of SS need to empower ethnic minority KTs to teach more flexibly and autonomously and offer more opportunities to develop their professional knowledge and skills by covering a wider range of population of ethnic minority KTs in order to enhance their SE in belief in completing the assigned task successfully and further improving their JS.

Moreover, the mediation role of WE on the association between SS and JS is supported, totally accepting the third hypothesis (H3). This result provides evidence for the previous theoretic arguments (Meijman and Mulder, 1998; Wang and Eccles, 2012) and empirical findings (Orgambidez-Ramos and de Almeida, 2017; Nasurdin et al., 2018). As depicted by Miao (2011), there are positive correlations of perceived and received organizational support and JS with task performance, including work activity, dedication, and focus among Chinese employees in the steel corporation. This result is also consistent with the finding of Yadav and Rangnekar (2015), disclosing that the support from supervisors promotes employees’ proactivity of participating in organizational decision-making behavior and JS. More importantly, this result verifies the Job Demands–Resources (JDR) model that posits that WE functions as a mediator in the association between job resources (e.g., SS) and organizational outcomes (e.g., JS) (Orgambidez-Ramos and de Almeida, 2017). Therefore, SS from various resources can inspire employees’ work performance and increase their level of WE (Shahpouri et al., 2016) and, consequently, the levels of JS through a mediation process (Schaufeli and Bakker, 2004b; Crawford et al., 2010). Underpinning these studies, we noted that mainland Chinese ethnic minority KTs’ JS might be directly promoted by enhancing SS and indirectly and cumulatively strengthened by the mediation of WE.

Finally, the result related to the last hypothesis (H4) reveals that SE has a positive effect on WE. In other words, SE mediates the association between SS and WE. At the same time, WE mediates the correlation between SE and JS. This result can be supported by the aforementioned theoretical discussion (Schaufeli and Salanova, 2010) and empirical findings (Llorens et al., 2007; Xanthopoulou et al., 2007). This result also gives support to the SCT of SE and SDT of WE, underlining that SE determines WE, acting as a self-motivation mechanism in JS (Høigaard et al., 2012; Chan et al., 2020). Mainland Chinese ethnic minority KTs with strong sense of SE tend to perform more engaged at work and resultantly those with high levels of WE are more inclined to be satisfied with job. Therefore, the chain mediation models are established in which the amount of SS mainland Chinese ethnic minority KTs receive presents opportunities to assess the extent to which they perceive the efficacy with which they engage with their work, with the consequent repercussion on their JS. As pointed out earlier, despite a few studies examining the link between SS, SE, WE, and JS in various sectors (Borgogni et al., 2013; Nasurdin et al., 2018), those studies omitted the educational sector, especially kindergarten institutes in remote areas. Regarding the analytical method, the multiple mediation model of SE and WE on the relationship between SS and JS has not previously been constructed. Therefore, this study has made an important contribution to current knowledge by advancing theoretical framework from a comprehensive perspective and offering empirical evidence from a specific group. As a country with 56 highly diverse ethnic groups, the research on the SS available for ethnic minority KTs as well as their efficacy, WE, and JS are tremendously worthwhile and representative. This study can be used as an example for other multi-ethnic countries aiming to promote educational equity, advance KTs’ work achievement and improve work well-being and health.

While reliability and validity issues of the construct were verified in the present study, limitations need to be acknowledged when assessing the results. One limitation is that the participants come from 17 ethnic minority groups (approximately 30% of 56 ethnic groups), which constrains the generalization of the findings; thus, further studies should include more ethnic minority KTs. Another limitation is that we do not consider the differences of all demographical variables that may result in statistical bias; therefore, future works should incorporate more controlled variables, such as gender, degree, and experience, to enhance the statistical power and the model discrimination.

## Conclusion

This study’s objective was to design a chain mediating model in which SS, SE, WE, and JS among mainland Chinese ethnic minority KTs were tested drawing on the SET, SCT, and SDT. The results reveal that SS from governments, organizations, and institutes is a pivotal antecedent of predicting SE, WE, and JS. The results also reveal that WE mediates the relationship between SS and JS and between SE and JS. In contrast, SE merely mediates the association between SS and WE. Although three posited constructs are accepted, and one is rejected, the proposed multiple mediation constructs are totally supported. This empirical result adds value to the current literature regarding the research on KTs’ job resources and job accomplishment from a perspective of ethnic groups. It also provides an informative insight into how to accelerate ethnic minority KTs’ SE, boost their WE, and ultimately, snowball their JS by offering more and higher quality SS from both cultural psychology and financial material and opportunities to facilitate their professional knowledge and skills for policymakers, governors, sponsors, supervisors, and other providers. This study can be further strengthened and generalized through extending the sample diversity to increase effect size and decrease random errors.

## Data Availability Statement

The original contributions presented in the study are included in the article/supplementary material. Further inquiries can be directed to the corresponding authors.

## Ethics Statement

The studies involving human participants were reviewed and approved by the College of Vocational and Technical Education, South China Normal University Human Research Ethics Committee. The patients/participants provided their written informed consent to participate in this study.

## Author Contributions

WC contributed to data curation. SW and MH contributed to conceptualization. SW and GF contributed to formal analysis. MH and HZ contributed to funding acquisition. WZ contributed to investigation. SW contributed to methodology. WC, HZ, and XL contributed to project administration. XL, XZ, and KZ contributed to resources. SW and HZ contributed to software. MH contributed to supervision. SZ and XY contributed to validation. SYW contributed to visualization. SW and XY contributed to writing—original draft. SW, XY, WC, WZ, MH, HZ, XL, and GF contributed to writing—review and editing. All authors contributed to the article and approved the submitted version.

## Conflict of Interest

The authors declare that the research was conducted in the absence of any commercial or financial relationships that could be construed as a potential conflict of interest.
